# Brain signal variability as a window into the bidirectionality between music and language processing: moving from a linear to a nonlinear model

**DOI:** 10.3389/fpsyg.2013.00984

**Published:** 2013-12-30

**Authors:** Stefanie Hutka, Gavin M. Bidelman, Sylvain Moreno

**Affiliations:** ^1^Department of Psychology, University of TorontoToronto, ON, Canada; ^2^NeuroEducation across the Lifespan Laboratory, Rotman Research Institute, Baycrest Centre for Geriatric CareToronto, ON, Canada; ^3^Institute for Intelligent Systems, University of MemphisMemphis, TN, USA; ^4^School of Communication Sciences and Disorders, University of MemphisMemphis, TN, USA

**Keywords:** musical training, tone language, transfer effects, nonlinear dynamical systems, brain signal variability

## Abstract

There is convincing empirical evidence for bidirectional transfer between music and language, such that experience in either domain can improve mental processes required by the other. This music-language relationship has been studied using linear models (e.g., comparing mean neural activity) that conceptualize brain activity as a static entity. The linear approach limits how we can understand the brain’s processing of music and language because the brain is a nonlinear system. Furthermore, there is evidence that the networks supporting music and language processing interact in a nonlinear manner. We therefore posit that the neural processing and transfer between the domains of language and music are best viewed through the lens of a nonlinear framework. Nonlinear analysis of neurophysiological activity may yield new insight into the commonalities, differences, and bidirectionality between these two cognitive domains not measurable in the local output of a cortical patch. We thus propose a novel application of brain signal variability (BSV) analysis, based on mutual information and signal entropy, to better understand the bidirectionality of music-to-language transfer in the context of a nonlinear framework. This approach will extend current methods by offering a nuanced, network-level understanding of the brain complexity involved in music-language transfer.

## INTRODUCTION

Within the last 30 years, the field of auditory cognitive neuroscience has started to compare the neurophysiological processing of music and language, finding evidence for shared, interactive processing in the neural regions that govern these domains (Besson and Macar, 1987; Maess et al., 2001; Koelsch et al., 2002; Patel, 2008; Slevc et al., 2009; Bidelman et al., 2011a). For example, the processing of musical features (e.g., melody and harmony) activate brain regions traditionally associated with language-specific processes, including the recruitment of Broca’s and Wernicke’s area, and the elicitation of certain electrophysiological markers, such as the N400 and P600 (Patel et al., 1998; Maess et al., 2001; Koelsch et al., 2002). In addition, neural regions traditionally associated with higher-order language comprehension (i.e., frontal areas, such as Brodmann Area 47) are active when trained musicians process complex musical meter and rhythm (Vuust et al., 2006).

These findings corroborate Patel’s (2011) OPERA hypothesis, which is a neurocognitive model that describes how music and language may benefit one another through shared, interactive processing between domains. Specifically, the OPERA (Overlap, Precision, Emotion, Repetition, Attention) framework outlines how the coordinated plasticity of musical training facilitates linguistic processing by recruiting overlapping language structures (e.g., Broca’s area) and increasing neural precision with these brain regions after emotionally driven, repetitive, and attentional engagement with music. The OPERA hypothesis was predicated on the *shared syntactic integration resource hypothesis* (Patel, 2003), which claimed that music and language rely on shared, limited processing resources, and that these resources activate separable syntactic representations. Collectively, this literature – both neurophysiological and theoretical – supports the notion of a shared neural mechanism underlying the melodic and rhythmic properties of both music and language, and illustrates the interactive processing between these domains.

### EVIDENCE FOR TRANSFER EFFECTS BETWEEN MUSIC AND LANGUAGE

Similarities in structure and processing demands between music and language raise the question if experience and learning in one of these domains can benefit processing in the other (i.e., transfer), and vice versa. At a theoretical level, the components of the OPERA model facilitate such transfer by postulating an experience-dependent enhancement to the neural processing (i.e., “precision”) for behaviorally relevant acoustic information – music, language, or otherwise. Such a mechanism may account for at least some of the linguistic benefits observed with musical experience. Yet, certain forms of language expertise also satisfy the components of the OPERA model, suggesting that it can also afford benefits in the neural processing of salient acoustic information. Indeed, it has been widely posited that musical training or certain language backgrounds may similarly contribute to cross-domain cognitive transfer (e.g., Bialystok and Depape, 2009; Moreno, 2009; Bidelman et al., 2011a, 2013; Moreno and Bidelman, 2013).

Ample empirical evidence supports transfer in the direction from music to language, ranging from the sensory-perceptual to the cognitive. Musical training has been associated with sensory-perceptual advantages in a number of language-specific abilities, such as phonological processing (Anvari et al., 2002), verbal memory (Chan et al., 1998; Franklin et al., 2008), verbal intelligence (Moreno et al., 2011), formant and voice pitch discrimination (Bidelman and Krishnan, 2010), sensitivity to prosodic cues (Thompson et al., 2004), detecting durational cues in speech (Milovanov et al., 2009), degraded speech perception (Parbery-Clark et al., 2009b; Bidelman and Krishnan, 2010), second language proficiency (Slevc and Miyake, 2006; Marques et al., 2007), lexical tone identification (Delogu et al., 2006, 2010; Lee and Hung, 2008), and temporal processing (Sadakata and Sekiyama, 2011; Marie et al., 2012).

At a cognitive level of analysis, music training has been associated with enhancements in executive processing, including verbal memory (Chan et al., 1998), intelligence (Schellenberg, 2004, 2006, 2011), working memory (WM) (Bugos et al., 2007; Pallesen et al., 2010; Bidelman et al., 2013), and executive control (Bialystok and Depape, 2009; as reviewed in Moreno and Bidelman, 2013). Moreno et al. (2011), for example, found that after short-term computer training programs in either music or visual art, children in the music group exhibited enhanced performance on a measure of verbal intelligence, with 90% of the sample showing behavioral improvement (no changes were found in the visual art group). This short-term music training also led to improved performance in an executive-function task (a visual go/no-go task) and showed plasticity in a neural correlate of that performance (increased P2 amplitude in the ERPs). Collectively, these studies demonstrate that extensive musical training tunes auditory neural mechanisms, enabling more robust encoding and control of basic auditory and speech information at both the sensory-perceptual and cognitive levels. Such advantages are supported by a wealth of electrophysiological data at subcortical (Wong et al., 2007; Musacchia et al., 2008; Parbery-Clark et al., 2009a; Bidelman et al., 2011a) and cortical (Pantev et al., 2001) levels of processing.

### EVIDENCE FOR TRANSFER EFFECTS BETWEEN LANGUAGE AND MUSIC

Unlike the evidence for music-to-language transfer, evidence for language-to-music transfer has, until recently, remained scarce and conflicting (Schellenberg and Peretz, 2008; Schellenberg and Trehub, 2008; see Bidelman et al., 2013 for a discussion). Tentative links have been drawn between tone-languages, in which the use of pitch distinguishes lexical meaning (Yip, 2002), and absolute pitch, the ability to name a note without a reference pitch (Deutsch et al., 2006; Lee and Lee, 2010). For example, there is evidence demonstrating a high incidence of AP in tone-language speakers. Deutsch et al. (2006) found that approximately 53% of tone-language speakers (Mandarin) possessed AP, compared to approximately 7% of non-tone-language speakers.

In relation to this finding, Deutsch et al. (2006) posited that the higher rates of AP in tone-language speakers might be owed to the initiation of musical training during the critical period for language acquisition. Due to the preponderance of meaningful pitch in their native language, tone-language speakers learn to associate tones with meaningful verbal labels; when these individuals begin musical training, this may facilitate the mapping between musical tones and note names and hence the development of AP (Deutsch et al., 2006). Though studying tone-language speakers is a good model for comparisons with musicians due to both groups’ enriched pitch-acuity, the aforementioned relationship between tone-language speakers and absolute pitch is not altogether informative. As discussed in Levitin and Rogers (2005), whether or not one possesses absolute pitch is largely irrelevant to most musical tasks that require relative (not absolute) pitch judgements. Thus, what we can learn about music-language transfer from the links between tone language and absolute pitch seems limited.

Behavioral studies have revealed contradictory findings on tone-language speakers’ nonlinguistic pitch perception abilities, ranging from weak (Giuliano et al., 2011; Wong et al., 2012) to no enhancements (Stagray and Downs, 1993; Bent et al., 2006; Schellenberg and Trehub, 2008; Bidelman et al., 2011b). It is possible that the equivocal findings of language-to-music transfer have been due to limitations of these behavioral studies including overly heterogeneous groups (e.g., pooling listeners across multiple language backgrounds, Pfordresher and Brown, 2009) and the use of overly simplistic musical stimuli (Bidelman et al., 2011b).

At the neural level, certain forms of bilingualism including experience with a tone language (Mandarin Chinese: Bidelman et al., 2011a, b), or Spanish (Krizman et al., 2012) have been found to affect both neural encoding, and the perception of behaviorally relevant sound. Long-term experience with specific parameters of speech, namely duration, has also been shown to extend past the processing of non-speech sounds, resembling the effects of music expertise (Marie et al., 2012). Marie et al. examined French musicians, French non-musicians, and Finnish (a quantity language, for which duration is a phonemically contrastive cue) non-musicians to test the influence of linguistic expertise on pre-attentive and attentive processing of non-speech sounds. Linguistic background and musical expertise influenced both pre-attentive and attentive auditory processing, with better discrimination accuracy for tones in Finnish non-musicians and French musicians compared to French non-musicians. The brain’s pre-attentive detection of frequency deviants was greater in French musicians than in both non-musician groups; no group differences were found for intensity deviants, suggesting some specificity in the language-music transfer. Thus, musical expertise influenced neurophysiological processing of multi-dimensional features of the auditory signal (duration and frequency) whereas linguistic expertise (i.e., Finnish experience) influenced only durational processing in music – the most acoustically relevant parameter shared with Finnish.

Additional evidence for transfer from language expertise to non-linguistic domains comes from Wong et al. (2012), who found that amusics^1^ who were tone-language (Hong Kong Cantonese) speakers showed improved pitch perception ability compared to non-tone language-(Canadian French and English) speaking amusics. This enhanced ability occurred in the absence of differences in rhythmic perception and persisted after controlling for musical background and age. These findings suggest that language experience plays an important role in tuning musical pitch perception and that tone language experience helps maintain normal pitch abilities in people with amusia. However, Nan et al. (2010), Jiang et al. (2010) did not find this link between language and the tuning of musical pitch perception, positing instead that pitch deficits in amusics may be domain-general and the processing of musical pitch and lexical tones may share certain cognitive resources (Patel, 2003, 2008, 2012). Conversely, in non-amusics, Zatorre and Baum (2012) argued that pitch processing differs for music and speech, positing that there are two pitch-related processing systems: one for the fine-grained, accurate representation necessary for music, and one for the coarse-grained, approximate analysis sufficient for language.

Given the inconsistent literature on language-to-music transfer, Bidelman et al. (2013) designed a study to carefully test if language expertise truly confers benefits on musical tasks. Specifically, the study investigated whether tone-language speakers would show enhanced performance on measures of music processing (discrimination, speech, and pitch memory) as compared to English-speaking musicians and non-musicians. Cantonese was chosen as the linguistic pitch group because the tonal inventory of the language (i.e., level pitch patterns) closely mirrors the way pitch unfolds in music. Specifically, Cantonese consists of six contrastive tones, of which most are level pitch patterns, minimally differentiable based on pitch height (Gandour, 1981; Khouw and Ciocca, 2007). Furthermore, the proximity of Cantonese tones is approximately that of a semitone – the smallest distance between adjacent tones in Western classical music (Peng, 2006). Results showed convincing evidence of language-to-music transfer, such that Cantonese speakers’ performance on tasks of auditory pitch acuity and music perception was enhanced relative to English-speaking non-musicians, and even comparable to that of musicians. Differences were also observed in visuospatial and auditory WM between groups. Musicians demonstrated large enhancements in both forms of WM compared to non-musician controls. Cantonese participants showed less robust but measurable enhancements in auditory WM performance relative to non-musicians.

**FIGURE 1:**
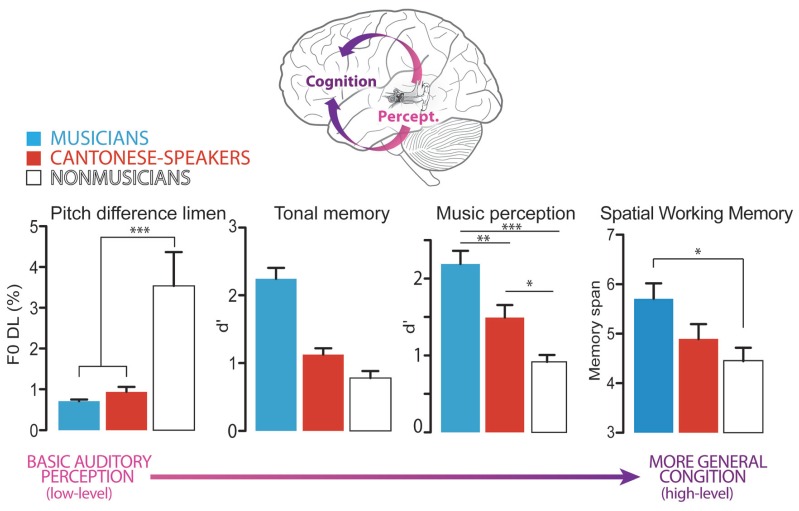
**Figure adapted from Bidelman et al. (2013), illustrating language-to-music transfer.** Enhanced perceptual and cognitive mechanisms operating in a processing hierarchy (from low-level auditory perception to more general cognition) may explain the behavioral and neural advantages observed in musicians versus tone –language bilinguals. Specifically, musicians and tone-language bilinguals show similar performance on auditory-perceptual tasks (e.g., pitch discrimination) but the groups diverge when considering more general cognitive dimensions (e.g., visuospatial WM). These data illustrate that while music-language transfer effects are bidirectional (both benefit one another), the magnitude of transfer is smaller in the language-to-music direction. *p < 0.05, **p < 0.01, ***p < 0.001.

These findings suggest that just as music can confer some advantages with language processing, language expertise can improve music listening abilities. Thus, there is a bidirectional relationship between music and language transfer. In addition, these findings suggest that tone-language bilinguals and musicians have similar performance on auditory-perceptual but not domain general cognitive dimensions (e.g., visuospatial WM). This suggests that the functional benefits of these two experiential factors begin to diverge when considering their benefits to high-order processes (**Figure 1**).

### UNDERSTANDING MUSIC-LANGUAGE TRANSFER AS A PERCEPTUAL-COGNITIVE HIERARCHY, AND LIMITATIONS OF CURRENT PERSPECTIVES

The findings of Bidelman et al. (2013) illustrate an important difference in how experience in music versus language tunes the brain. It is possible that music may induce more widespread effects than tone-language expertise. Thus, though functional similarities between music and language are revealed at a perceptual level when considering simple auditory tasks, differences begin to emerge at more cognitive levels, which govern more complex forms of analysis. This functional divergence may arise from nuanced differences in overlap for the networks involved in the processing of language and music. This perspective is in agreement with the complex relationship between music and language processing and the related difficulties in directly comparing the two domains. This relationship has been highlighted in several recent reviews (Kraus and Chandrasekaran, 2010; Besson et al., 2011; Moreno and Bidelman, 2013).

In an examination of the relationship between music training and the development of auditory skills, Kraus and Chandrasekaran (2010) explored the neural representation of pitch, timing, and timber in the human auditory brainstem. The authors posited that the effect of music training leads to fine-tuning of all salient auditory signals, both musical and non-musical (Kraus and Chandrasekaran, 2010). Further exploring these mechanisms of transfer, Besson et al. (2011) stated that when long-term experience in a domain impacts acoustic processing in another domain, the findings can serve as evidence for common acoustic processing. Similarly, when long-term experience in one domain influences the build-up of abstract and specific percepts in another domain, results may serve as evidence for transfer effects.

Extending this perspective to a more global approach, Moreno and Bidelman (2013) reconciled the sensory and cognitive benefits of musical training by positing a multidimensional continuum model of transfer. In this model, the extent of transfer and the neural systems affected by it are viewed as spectrum along two orthogonal dimensions, namely Near-Far and Sensory-Cognitive. The former describes the extent of transfer; the latter describes the level of affected processing, ranging from low-level sensory processing specific to the auditory domain to high-level domain-general cognitive processes, supporting executive function and language. The next step is to test this model at the neural level, to better examine how these different levels of transfer are manifested. However, a central challenge with this examination is teasing apart acoustic from abstract representations of processing, due to high-degree of interaction between acoustic versus abstract representations during speech perception and cognition (Besson et al., 2011). We posit that this challenge arises because of the linear approach applied to the study of music-language interactions. That is, empirical work on this relationship relies on methods such as mean activation in or between neural regions that cannot adequately characterize the complex cross-domain interactions observed at the behavioral level.

This disconnection between our understanding of these neural networks and behavioral findings is well-illustrated by studies that suggest that these networks are distinct, despite sharing a number of commonalities and interactions at the behavioral level. For example, there do not appear to be cumulative benefits of language *and* music expertise on auditory cognitive tasks, even in the presence of benefits afforded by each individual domain. Cooper and Wang (2012) engaged tone language (Thai) and non-tone language (English) speakers, subdivided into musician and non-musician groups, in Cantonese tone-word training. These participants were trained to identify words distinguished by five Cantonese tones and they completed tasks of musical aptitude and phonemic tone identification. Participants who spoke Thai and/or were musicians were better at Cantonese word learning. However, having both tone-language experience and musical training was not advantageous above and beyond either type of experience alone. This suggests that the networks underlying the processing of verbal tones in musicians and tone-language speakers confer similar but not cumulative behavioral benefits.

Similarly, Mok and Zuo (2012) investigated whether musical training had facilitatory effects on native tone language speakers. The authors had Cantonese and non-tone language speakers with or without musical training perform discrimination tasks with Cantonese monosyllables and pure tones resynthesized from Cantonese lexical tones. While musical training enhanced lexical tone perception for non-tone language speakers, it had little effect on the Cantonese speakers. Together with the results of the aforementioned studies, this suggests that mechanisms governing linguistic and musical processing belong to partially overlapping but not identical brain networks. Divergent patterns of activation within these networks may explain why there are no cumulative advantages conferred by possessing both music and language expertise. These networks may afford similar behavioral outcomes given appropriate environmental exposure (i.e., tone-language or music training) and task demands. Such an explanation would account for the lack of an additive beneficial effect on processing as well as differences in perceptual versus cognitive transfer between language and music (e.g., Bidelman et al., 2013). These studies collectively demonstrate that, despite aforementioned similarities, the networks that process each domain are separate at the behavioral level. By virtue of being different – but interacting – systems, a linear model lacks the capacity to disentangle music-language interactions at the neural level. This presents the need to understand this relationship using a *nonlinear* model.

Further supporting the shift to a nonlinear model is the observation that the brain itself is a complex nonlinear system (McKenna et al., 1994; Bullmore and Sporns, 2009), and requires a nonlinear model for greater explanatory power of its functions. We thus propose the application of a nonlinear, network-level analysis to facilitate the understanding of music and language processing. To this end, we discuss the brain as a nonlinear system, and how the neural processing of language and music signals can best be viewed through the lens of a dynamic, nonlinear systems approach using novel analysis techniques adopted from information theory and neuroimaging studies.

## THE BRAIN AS A COMPLEX, NONLINEAR SYSTEM

Complex nonlinear systems are typically characterized as dynamic (i.e., they change with time), nonlinear (i.e., the effect is disproportionate to the cause), multifaceted, open, unpredictable, self-organizing, and adaptive (Larsen-Freeman, 1997, p. 142). Dynamic systems have been addressed by theories including chaos and dynamic systems theory (Abraham, 1994). Complex systems have certain topological properties, including high clustering, small-worldness (the ability for a large network to be traversed by a small number of steps), the presence of high-degree nodes or hubs, and hierarchy (Bullmore and Sporns, 2009, p. 187). As Bullmore and Sporns (2009) highlight, these properties have been measured in brain networks (small-worldness, Sporns et al., 2004; Bassett and Bullmore, 2006; Reijneveld et al., 2007; Stam and Reijneveld, 2007; hierarchy, Ravasz and Barabási, 2003; centrality, Barthelemy, 2004; and distribution of network hubs, Guimera and Amaral, 2005). Furthermore, the behavior of a complex nonlinear system, such as the brain, does not emerge from any single component but instead from the interaction between its ever-changing constituent components (Waldrop, 1992, p. 145). Given the definition of the brain as a complex, nonlinear system, we posit that linear analyses of the brain cannot portray a complete account of neural functioning, and must be complemented with nonlinear techniques.

### NONLINEAR SYSTEMS AND THE EMBODIED MIND: THE BRAIN AS A “SIGNAL-PROCESSOR” OF MUSIC AND LANGUAGE

Several groups have started to use a nonlinear model of language and music processing to characterize the emergence of these two domains at the behavioral and neural level. Specifically, there is evidence that language (Larsen-Freeman, 1997; Vosse and Kempen, 2000; Tabor and Hutchins, 2004) and music (e.g., Large and Almonte, 2012) can be understood as complex nonlinear signals, with patterns that emerge over multiple timescales. Language, Larsen-Freeman (1997) posits, is both complex and nonlinear, such that language use (e.g., grammar) is dynamic and variable, subject to growth and change, and emerges in a non-incremental manner. The co-occurrence of words in sentences reflects language organizations that can be described in a graph of word interactions, to which small-world properties are applicable (i Cancho and Solé, 2001). Similarly, dynamical system models have been applied to syntax in language (Tabor, 1995; Cho et al., 2011), as well as music (Marin and Peltzer-Karpf, 2009).

As discussed by Large and Almonte (2012), the theory of nonlinear dynamical systems has also been applied to music tonality, explaining perception of consonance and dissonance in musical intervals (Lots and Stone, 2008) and tonal stability in musical melodies (Large and Tretakis, 2005; Large, 2010a, b). Specifically, the Large (2010a) dynamic theory of musical tonality predicts that, as auditory neurons resonate to musical stimuli, dynamical stability, and attraction arise among neural frequencies. These dynamics give rise to the perception of relationships among tones, collectively referred to as tonal cognition (Large and Almonte, 2012). Large and Almonte (2012) used this model of musical tonality to predict scalp-recorded human auditory brainstem responses elicited by musical pitch intervals. Modeled brainstem responses showed qualitative agreement with many of the central features of empirically recorded human brainstem potentials. In addition to tonality, the perception of metrical structure has been viewed as a dynamic process, where the temporal organization of external musical events synchronizes a listener’s internal processing mechanisms (Large and Kolen, 1994). Indeed, a nonlinear oscillator model driven with complex, non-stationary rhythms arising from musical performance has adequately modeled musical beat perception (Large, 1996). These functional, nonlinear models of tonality and beat perception support viewing aspects of music functioning as complex, nonlinear signals.

These nonlinear models of tonality and beat perception have important implications for understanding how we can model sensory inputs (e.g., using measures such as entropy) to facilitate embodied artificial intelligence (Sporns and Pegors, 2004), as well as better understand the embodiment of music in consciousness^2^. For example, dynamic changes in musical timing have been shown to predict both ratings of emotional arousal, as well as real-time changes in neural activity (Chapin et al., 2010). This emergent, temporal dimension of music elegantly aligns with the multiple time scales of analysis used in nonlinear methods, but not in linear methods.

### SHIFTING FROM A REDUCTIONIST TO A NONLINEAR APPROACH

**FIGURE 2:**
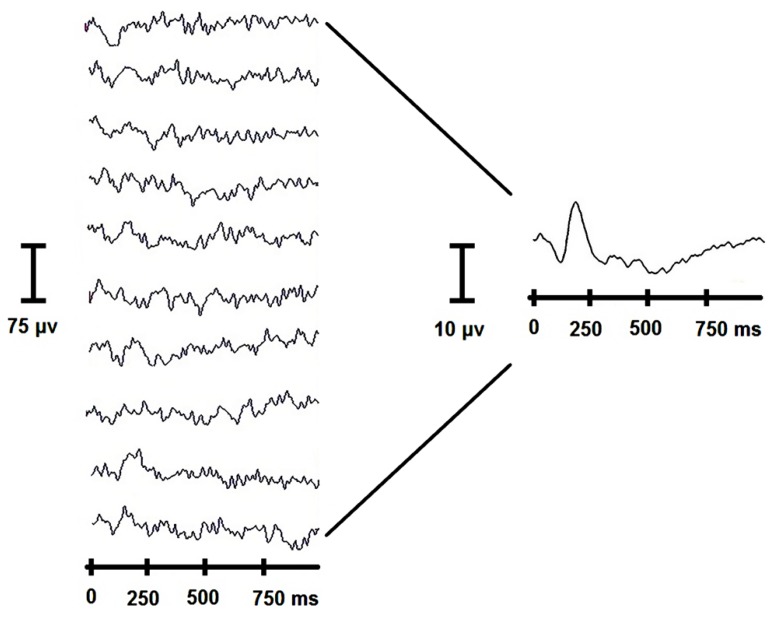
**Loss of information as a result of traditional linear analysis of the EEG.** The variation between individual trials (at left) is lost as a result of the averaging procedure, as evident in the averaged waveform (at right).

In light of the evidence that both music and language can be conceptualized as complex, nonlinear systems operating within a dynamical brain system, it seems pragmatic to shift from a reductionist view to a nonlinear approach. In the former, neural activity is studied as a static, local entity; in the latter, neural activity is studied by measuring brain signal variability (BSV), in which the entire neural network’s activation and interactions are considered. For example, in a linear approach to electroencephalography (EEG), waveforms are averaged together across trials. A loss of information is inherent to this process, as the variability in each trial disappears as a result of averaging (**Figure 2**). Using a nonlinear framework, one can capture this variability across time. As we will discuss, this variability can contain valuable information, and can characterize group-differences in a more nuanced way than a linear approach (e.g., mean responses). Thus, differences not revealed in a linear approach can be revealed via nonlinear methods.

We thus posit that the nonlinear approach would reveal a rich relationship between the networks recruited as a result of music training and language expertise, specifically in the subcortical and cortical brain regions identified to promote transfer (e.g., Maess et al., 2001; Koelsch et al., 2002; Bidelman et al., 2011a, b, c) and could potentially explain some of the mechanisms underlying the bidirectionality of transfer in music and language. As a means of investigating the complexity of neural networks underlying the bidirectionality phenomenon, we propose a novel application of the BSV framework. Through BSV analyses, we can go one step beyond modular views of music and language processing and brain functional organization, to reveal the dynamic interactions between the complex, nonlinear systems of music and language.

## BRAIN SIGNAL VARIABILITY: DEFINITION AND METHODS

In the complex nonlinear system that is the brain, we find inherent variability (Pinneo, 1966; Traynelis and Jaramillo, 1998; Stein et al., 2005; Faisal et al., 2008), fluctuating across time, both extrinsically (i.e., during a task, Raichle et al., 2001; Raichle and Snyder, 2007; Ghosh et al., 2008; Deco et al., 2009, 2011) and intrinsically (i.e., at rest, Deco et al., 2011). Functional connections emerge and dissolve over time, giving rise to cognition, producing signals with high variability. As discussed in Faisal et al. (2008), variability arises from two sources – the deterministic properties of a system (e.g., the initial state of neural circuitry will vary at the start of each trial, leading to different neuronal and behavioral responses), and “noise,” which are disturbances that are not part of the meaningful brain activity and thus interfere with meaningful neural representations. We are referring to the former type of variability, that which reflects important brain activity and not, for example, random artifacts inherent to the acquisition of brain data [e.g., ocular/muscular perturbations or thermal noise from electrodes or magnetic resonance imaging (MRI) scanners]. This BSV is the transient temporal fluctuations in brain signal (Deco et al., 2011; see Garrett et al., 2013a for BSV formulae); its analysis can be applied to many different types of neuroimaging data.

For example, BSV has been analyzed in EEG (McIntosh et al., 2008, 2013; Lippé et al., 2009; Protzner et al., 2010; Heisz et al., 2012), functional magnetic resonance imaging (fMRI; Garrett et al., 2010, 2011) and MEG (Misić et al., 2010; Vakorin et al., 2011; Raja Beharelle et al., 2012; McIntosh et al., 2013). In the EEG study by McIntosh et al. (2008), BSV was examined using two measures, namely principal component analysis (PCA, a linear method which was applied here in a nonlinear way) and multiscale entropy (MSE, a nonlinear metric). These measures prove sensitive to linear and nonlinear brain variability and differentiate between changes in the temporal dynamics of a complex system and that of random variability (Costa et al., 2002, 2005). MSE indexes the temporal predictability of neural activity, calculated by downsampling single-trial time series to progressively coarse-grained time scales, and calculating sample entropy (i.e., state variability) at each scale (Costa et al., 2005). Such a linear versus nonlinear differentiation would be useful in qualifying the complexity of complementary neural networks – particularly, temporally sensitive networks such as those responsible for language and music processing.

Recently, BSV has been found to convey important information about network dynamics, such as integration of information (Garrett et al., 2013b) and comparing long-range versus local connections (McIntosh et al., 2013). That is, BSV can serve to reveal a complex neural system that has capacity for enhanced information processing and can alternate between multiple functional states (Raja Beharelle et al., 2012). BSV thus affords the appropriate framework with which the interaction of music and language can be studied, allowing us to view these two systems as dynamically fluctuating across time.

As discussed in Garrett et al. (2013b), the modeling of neural networks involves mapping an integration of information across widespread brain regions, via emerging and disappearing correlated activity between areas over time and across multiple timescales (Jirsa and Kelso, 2000; Honey et al., 2007). These transient changes result in fluctuating temporal dynamics of the corresponding brain signal, such that more variable responses are elicited by networks with more potential configurations or “brain states” (Garrett et al., 2013b). This signal variability is thought to represent the network’s information-processing capacity, such that variability is positively associated with integration of information across the network (Garrett et al., 2013b). Thus, this variability is experience-dependent (rather than task-dependent), making such representations a valuable addition to understanding the interaction of neural mechanisms supporting music and language behaviors.

### APPLICATION OF BSV TO UNDERSTANDING PERCEPTUAL PROCESSES

The analysis of BSV from EEG, MEG, and fMRI is a new framework in cognitive neuroscience data analysis. Of the existing literature on BSV, several studies have focused on developmental applications of BSV. For example, McIntosh et al. (2008) studied the relationship between variability in single-trial evoked electrical activity of the brain (measured by EEG) and performance on a face memory task in children (age 8–15) and young adults (ages 20–33). Both PCA and MSE analyses revealed that EEG signal variance increased with age. Furthermore, behavioral stability, measured by accuracy and intra-subject variability of reaction time, increased as a function of greater BSV.

This finding was confirmed by Lippé et al. (2009) in children aged one-month to 5 years. These results support a relationship between increased long-range connections and maturation from childhood to adulthood. This is an important application for the understanding of the bidirectionality of transfer between language and music because it allows us to explore the link between these two domains at both the scope of long-range functional connections and local processing. This ability would allow for the understanding of transfer between the domains of language and music along a spectrum ranging from regional effects of music training (affecting regions involved in language processing) to more long-range network changes (e.g., fronto-temporal coupling). This would align with the aforementioned model by Moreno and Bidelman (2013), which conceives music-language transfer as a multidimensional continuum of two, orthogonal dimensions: the level of affected processing (ranging from low-level sensory to high-level cognitive transfer) and the distance of transfer from the domain of training (ranging from near to far).

Misić et al. (2010) replicated the findings of McIntosh et al. (2008) over a larger age range (6–16 and 20–41 years) using a different neuroimaging method, namely MEG. Using BSV, Misic et al. found that during development, neural activity became more variable across the entire brain, with the most robust increases seen in medial parietal regions. As these are regions previously shown to be important for integration of information from different areas of the brain, the authors suggested that within BSV, one can observe transient changes in functional integration that are modulated by task demand. Such an observation would be integral to understanding how networks, respectively involved in music and language are integrated, and how this integration is related to behavioral performance.

The application of BSV to fMRI has also proven highly informative in understanding brain networks. Garrett et al. (2010) found that the standard deviation of BOLD signal was five times more predictive of brain age (from age 20 to 85) than mean BOLD signal. In another study, Garrett et al. (2011) examined how BOLD variability related to age, reaction time speed, and consistency in healthy younger (20–30 years) and older (56–85 years) adults on three cognitive tasks (perceptual matching, attentional cueing, and delayed match-to-sample). Younger, faster, and more consistent performers exhibited increased BOLD variability, establishing a functional basis for this often disregarded measure. These studies collectively demonstrate the importance of shifting from a linear (e.g., mean neural response) to a nonlinear (e.g., entropy/variability) conception of complex brain systems and their relationship to behavior.

Brain signal variability has also been applied to the study of knowledge representation by Heisz et al. (2012). Heisz et al. tested whether BSV reflects functional network reconfiguration during memory processing of faces. The amount of information associated with a particular face was manipulated (i.e., the knowledge representation for each face; for example, a famous face would have more information associated with it, and thus, greater knowledge representation), while measuring BSV to capture the EEG state variability. Across two experiments, Heisz et al. found greater BSV in response to famous faces than a group of non-famous faces, and that BSV increased with face familiarity. Heisz et al. posited that cognitive processes in the perception of familiar stimuli may engage more widespread neural regions, which manifest as higher variability in spatial and temporal brain dynamics.

The findings of Heisz et al. corroborate those of Tononi et al. (1996), who found that the amount of information available for a given stimulus can be determined by the extent to which the complexity of a stimulus matches its underlying system complexity. For example, familiar stimuli would elicit a stronger match than novel stimuli, as there would be more information available on the former, thus yielding greater BSV. These findings collectively suggest that BSV increases as a result of the increased accumulation of information within a neural network. Presumably, this type of “build-up” results from the increased repertoire of brain responses associated with a given stimulus (Tononi et al., 1994; Ghosh et al., 2008; McIntosh et al., 2008). These findings are applicable to understanding the bidirectionality of music and language at a network level because brain responses associated with given stimuli (i.e., differences between musical notes or lexical tones) should commensurately vary in BSV for a group that has expertise with those stimuli (i.e., musicians or tone-language speakers).

### A NOVEL APPROACH: USING BRAIN SIGNAL VARIABILITY TO UNDERSTAND THE BIDIRECTIONALITY OF MUSIC AND LANGUAGE

As discussed earlier, traditional approaches to understanding the brain (e.g., fMRI: mean activation; ERPs: peak amplitudes) do not afford a complete understanding of the brain given that they disregard inherent nonlinear processing and state variability. Nonlinearity may explain why we observe perceptual or cognitive differences in music-language transfer (e.g., Bidelman et al., 2013), distinct functional differences despite overlapping structures subserving song and speech (i.e., Tierney et al., 2013), and non-cumulative effects of music and language expertise (i.e., Cooper and Wang, 2012; Mok and Zuo, 2012). A network-level framework may better represent the behavioral manifestations of the transfer (and/or uniqueness) between music and language. In addition, this framework will allow us to move beyond modular views of language and music processing toward a more global conceptualization of functional brain organization.

This leads us to posit some predictions of what BSV might reveal about the music-language relationship. We have evidence that experience in a given domain enriches information integration and knowledge representation for domain-specific stimuli (Tononi et al., 1996; McIntosh et al., 2008; Raja Beharelle et al., 2012; Heisz et al., 2012; Garrett et al., 2013b). Thus, we might predict that this enrichment would be reflected in increased BSV and increased functional capabilities (i.e., the brain is more variable because transient networks form and dissolve, facilitating a greater behavioral repertoire). Similarly, for cross-domain transfer effects, one might predict increased BSV in response to stimuli belonging to a complementary domain of that transfer, as compared to stimuli in an unrelated domain (e.g., visual stimuli). This is particularly relevant to the future study of transfer in expert populations. For example, in the case of music-to-language transfer, one might predict high BSV and behavioral stability in the EEG data of a musician collected in response to distinguishing between vowel sounds but not visual objects. Between music- and language-expert groups, one might observe differences from linear analyses (e.g., mean EEG amplitude) but similar BSV in response to music and language stimuli. This would indicate similarity in *network function* between domains, while maintaining *distinct representations* between domains. Alternatively, one might not observe between-group differences in traditional measures, such as behavior and/or evoked response mean amplitudes and latencies, but see differences in BSV in response to music and language stimuli. This may be due to network-level differences in these domains and might reveal the extent of bidirectionality between them. Such findings would support the view of functionally similar but distinct networks for language and music. Such dissociations offer a new angle from which to examine the bidirectionality of music-to-language transfer, which could be used in conjunction with traditional neuroimaging analyses.

Ongoing work in our lab is beginning to explore BSV in examining bidirectional transfer effects associated with music and language expertise. Furthermore, experience in a given domain (e.g., music or language) is associated with integrated knowledge representation within that domain, reflected in more stable behavioral responses to such stimuli. This integrated knowledge representation would be reflected in increased BSV which, in turn, would be associated with increased stability in expert-groups’ respective behavioral responses. Differential outcomes are predictable for musicians versus a language-expert group. For example, comparing trained musicians to tone-language speakers, we would expect a general enhancement but differential activation in a given brain network associated with the processing of music vs. speech stimuli. This would illustrate experience-dependent effects from expertise in either domain (e.g., higher BSV for music and language stimuli relative to controls) but a specificity due to the unique (domain-specific) knowledge representation. While these outcomes remain hypothetical for the moment, they provide a testable framework for future experiments exploring the neurophysiological effects of language and music experience.

## CONCLUSION

We have reviewed evidence to support bidirectionality of language and music transfer, specifically demonstrating that music and language share similar networks in the brain and confer similar but not identical functions. However, we also identified some discrepancies which point out the complexity of the language-music relationship. Our review identifies the limitations of a linear model in understanding music and language and has proposed that music and language are best understood in a framework of integrative, nonlinear dynamical systems rather than a series of static neural activations. This shift toward a nonlinear model is supported by evidence for different, interactive networks supporting music and language processing in a nonlinear manner and, most importantly, by the view of the brain as a nonlinear system.

We have proposed a new approach to facilitate the understanding of music and language processes, namely the exploration of BSV, and advocate a shift from a linear to a nonlinear framework to more fully understand the neural networks underlying language and music processing. BSV has the potential of constructing such a dynamical neural model of music and language processing, as well as the transfer between these domains. Specifically, through BSV, we can understand how perceptual and cognitive mechanisms operate in a processing hierarchy, from low-level auditory perception to more general cognition, at both the behavioral and neural level. BSV can not only complement traditional methodological approaches, but also provide novel insights to brain organization and transfer between various cognitive functions.

## Conflict of Interest Statement

The authors declare that the research was conducted in the absence of any commercial or financial relationships that could be construed as a potential conflict of interest.

## ACKNOWLEDGMENTS

We would like to thank Sean Hutchins, Sarah Carpentier, and Patrick Bermudez for their helpful comments on earlier versions of this manuscript. We would also like to thank James Marchment for his valuable assistance with illustrating the figures. This work was financially supported through grants awarded to Stefanie Hutka from the Natural Sciences and Engineering Research Council of Canada (NSERC): create in Auditory Cognitive Neuroscience, and Sylvain Moreno from the Federal Economic Development Agency for Southern Ontario (FedDev Ontario).

## Footnotes

1 Congenital amusia is a neurogenetic disorder affecting music (pitch and rhythm) processing that affects approximately 4–6% of the Western (non-tone language speaking) population (Kalmus and Fry, 1980; Peretz etal., 2008).

2 See Thompson and Varela (2001) for a discussion on two-way relationships between embodied conscious states and local neuronal activity.
